# Stage IIB-IVA cervix carcinoma in elderly patients treated with radiation therapy: a longitudinal cohort study by propensity score matching analysis

**DOI:** 10.1186/s12905-023-02427-8

**Published:** 2023-05-17

**Authors:** Huafeng Shou, Qiuyan Wan, Hong’en Xu, Lei Shi, Tao Song

**Affiliations:** 1 Department of Gynecology, Zhejiang Provincial People’s Hospital, Affiliated People’s Hospital, Hangzhou Medical College, Hangzhou, 310014 Zhejiang People’s Republic of China; 2 Department of Gynecologic Oncology, Jiangxi Cancer Hospital, Nanchang, 330029 Jiangxi People’s Republic of China; 3 Cancer Center, Department of Radiation Oncology, Zhejiang Provincial People’s Hospital, Affiliated People’s Hospital, Hangzhou Medical College, Zhejiang 310014 Hangzhou, P.R. China; 4 Cancer Center, Department of Radiation Oncology, Zhejiang Provincial People’s Hospital, Affiliated People’s Hospital, Hangzhou Medical College, No. 158, Shangtang Road, Gongshu District, Hangzhou, 310000 P. R. China

**Keywords:** Cervical Cancer, Age at diagnosis, SEER, Overall survival, Radiotherapy

## Abstract

**Objective:**

We aimed to evaluate the treatment modality and prognostic impact of the age at diagnosis on stage IIB-IVA cervix carcinoma (CC) patients who received radiotherapy (RT).The evaluation was performed using the Surveillance, Epidemiology, and End Results (SEER) database.

**Patients and methods:**

From the SEER database, we included the patients with a histopathological diagnosis of CC between 2004 and 2016. Subsequently, we compared the treatment outcomes between patients aged ≥ 65 years (OG) and < 65 years (YG) by propensity score matching (PSM) analysis and Cox proportional hazard regression models.

**Results:**

The data of 5,705 CC patients were obtained from the SEER database. We observed that the OG patients were significantly less likely to receive chemotherapy, brachytherapy, or combination treatment compared to the YG (*P* < 0.001). Further, the advanced age at diagnosis was an independent prognostic factor associated with decreasing overall survival (OS) before and after PSM. Even in the subgroup analysis of patients who received trimodal therapy, an advanced age had a significant negative impact on OS compared to their younger counterparts.

**Conclusion:**

Advanced age is associated with less aggressive treatment regimens and is independently associated with impaired OS for stage IIB-IVA CC patients who received RT. Hence, future studies should incorporate geriatric assessment into clinical decision-making to select appropriate and effective treatment strategies for elderly CC patients.

**Supplementary Information:**

The online version contains supplementary material available at 10.1186/s12905-023-02427-8.

## Background

Cervical carcinoma (CC) is the fourth most incident cancer with a high mortality rate among female cancer patients globally (GLOBOCAN). In 2020, 341,831 cancer-related deaths worldwide were due to CC [1]. The International Federation of Gynecology and Obstetrics (FIGO) stage IIB-IVA cancer accounts for more than two-thirds of all CC patients at initial diagnosis and over 80% of all cancer patients in developing countries [2, 3]. The FIGO and the National Comprehensive Cancer Network (NCCN) guidelines recommend the definitive concurrent chemoradiotherapy (CCRT) based on cisplatin with brachytherapy (BRT) as the standard treatment option for CC [4–6]. Some studies from the early 2000s have estimated that by 2030, elderly patients (≥ 65 years) will represent approximately 20% of the US population but will comprise over 70% of all cancer patients [7, 8]. However, dealing with this soon-to-erupt problem and appropriately applying the standard treatment regimens for elderly patients is a great challenge in clinical settings.

Due to vast heterogeneity among the elderly CC patients, they are excluded from most clinical trials. Hence, evidence regarding the management of elderly CC patients mostly came from retrospective cohort studies [9–11]. In a propensity score-matching (PSM) study from Taiwan, the overall survival (OS) was compared between patients over 75 years and less than 60 years [12]. A significant difference in survival was documented in the 5-year OS between the elderly and younger group after PSM (49.2% versus 71.5%, respectively, *P* < 0.001). Additionally, the 5-year cumulative incidence rate of grade 2 (39.7% (elderly group) versus 17.2% (younger group)) and grade 3 (18.1% (elderly group) vs. 6.2% (younger group)) radiation-induced proctitis was significantly higher in the elderly group than in the younger group (*P* = 0.015 and 0.040, respectively). Moreover, in a large-sample single-institutional study, Ikushima and his colleagues evaluated the therapeutic efficiency of radical radiotherapy (RT) for older CC patients and compared its survival outcomes with younger patients (< 65 years). However, contrary to the previous study, they demonstrated that RT was well tolerated in elderly CC patients. Further, the 5- and 10-year disease-specific survival rates of patients between 65 and 74 years were significantly superior to the survival rates of younger patients (*P* < 0.001). Further, a multivariate Cox proportional hazard model demonstrated that advanced age is a non-significant prognostic factor of CC [13]. Similar findings were also reported in other studies [14, 15].

Thus, the prognostic importance of the diagnostic age on the survival of CC patients remains uncertain. We believe that a well-designed retrospective study might address the question of the association between the diagnostic age and CC survival, which will ensure better management of elderly CC patients. Hence, in this study, we aimed to explore the treatment modalities and prognostic impact of the age at diagnosis on the OS of elderly CC patients with stage IIB-IVA diseases who received RT and/or chemotherapy (CT). Further, we compared the survival outcomes of patients aged < 65 years using data from the Surveillance, Epidemiology, and End Results (SEER) database through the PSM analysis.

## Materials and methods

### Data collection and selection criteria

**Fig. 1:**
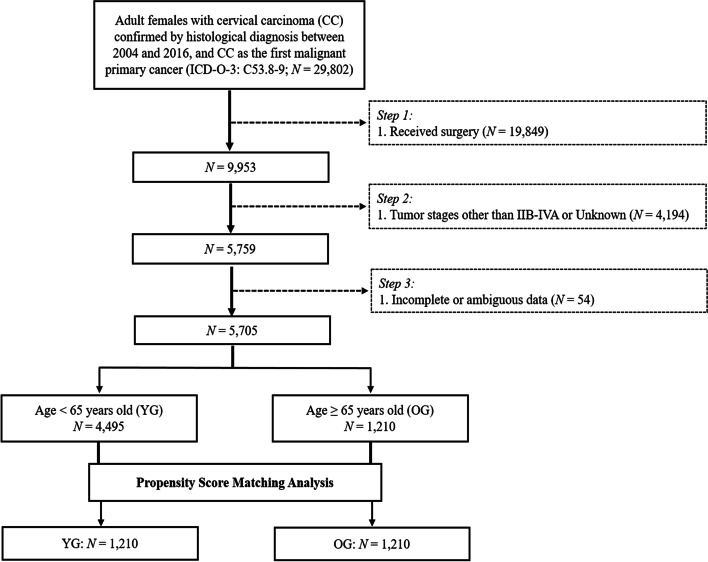
Patient selection flowchart

We used SEER*Stat 8.3.6 software (username for log in: 10,579-Nov2019) to retrieve stage IIB-IVA CC patients from 2004 to 2016 under the International Statistical Classification of Diseases for Oncology, 3rd Edition (ICD-O-3) recodes of C53.8–9. Inclusion criteria included the following: 1) patients with a confirmed histopathological diagnosis of CC; 2) primary diagnosis of CC with IIB–IVA staging; 3) patients who received at least either external beam RT (EBRT) alone or EBRT plus BRT as clearly indicated in the SEER program. Patients with incomplete or ambiguous data were excluded and the selection flow chart is presented in Fig. 1.

### Data processing

After filtering data extracted from the database, the following variables were employed in the subsequent analysis: patient characteristics (age at diagnosis, race, and marital status), tumor characteristics (histology type, grade, tumor stage, and tumor size), treatment characteristics (RT and CT), and survival data, including survival status and survival time in months. We defined race as a ternary variable as white, black, and others, which included American Indian/Alaska Native, Asian, Pacific Islander or unknown [16]. Tumor grade was also assorted as a ternary factor and categorized based on tumor differentiation into well or moderately differentiated, poorly or undifferentiated, and unknown [17]. Marital status, histology, and CT were defined as binary factors, as adopted in other SEER studies [18–20]. Tumor stages were initially registered based on the American Joint Committee on Cancer (AJCC) TNM 6^th^ edition for patients diagnosed between 2004 and 2015, and SEER database combined stage for patients registered in 2016 within the original data. Tumors were then restaged based on the 2014 FIGO staging system [21] since our previous SEER study have observed non-superior prognostic influence of the 2018 FIGO staging system over the 2014 FIGO staging system [22] and this issue had also been raised in other reports [23, 24].

### Outcome and statistical analysis

Given that locally advanced CC patients could enjoy a long-term survival time with appropriate treatments, OS was selected as the primary endpoint based on previously published perspectives [25, 26], and OS was determined as the duration from the diagnosis of CC to the time of death or the last follow-up. Baseline characteristics of enrolled patients were summarized by descriptive statistics and frequency tables. The chi-square test analysed the differences in baseline characteristics. To reduce selection bias of baseline characteristics between the two groups, we performed a PSM analysis at a ratio of 1:1 for covariates, including marital status, race, tumor size, 2014 FIGO stage, RT, and CT by the R “*MatchIt*” package. The PSM analysis was performed as described in our previous studies [27, 28]. Survival curves were estimated using the Kaplan–Meier analysis and compared with the log-rank test. The prognostic value of different variables was identified through univariate and multivariate Cox regression models with a hazard ratio (HR) and 95% CI (confidence interval) analyses. Differences were considered significant if the two-sided *P*-values were less than 0.05.

Statistical analyses were performed by the Statistical Package for the Social Sciences (SPSS) software, version 25.0 (IBM Corporation, Armonk, NY, USA) and the R software, version 3.6.2 (https://www.r-project.org, Institute for Statistics and Mathematics, Vienna, Austria). Survival curves were drawn using the GraphPad Prism 8.0 (GraphPad Software, San Diego, CA, USA).

## Results

### Baseline characteristics

Overall, a total of 5,705 primary CC patients with stage IIB-IVA diseases who received RT between 2004 and 2016 were identified from the SEER registry. 4,061 (80.0%) patients were white and squamous cell carcinoma (SCC) was the predominant type of cancer. The median size of known masses was 60 mm with 395 (7.8%) patients had tumors less than 40 mm. Based on the study design, we divided the enrolled patients into two groups — those whose age at diagnosis was less than 65 years (YG, young group) and those whose age at diagnosis was more than or equal to 65 years (OG, old group). The baseline characteristics of these patients were demonstrated in Table 1. Compared to the YG patients, the OG patients received significantly less BERT plus BRT, and treatment of CT (both *P* < 0.001). Given the significant differences in the marital status, race, tumor size, FIGO stage, RT, and CT between the YG and OG patients, a PSM analysis was applied to balance the distribution of these baseline characteristics. After matching, no demographic and clinical characteristics were significantly different between the two groups.

**Table 1:** Baseline characteristics of enrolled patients before and after PSM

Characteristic	Before matching			After matching		
	< 65 (n, %)	≥ 65 (n, %)	*P* value	< 65 (n, %)	≥ 65 (n, %)	*P* value
*Marital status*			< 0.001			0.614
Married	1674 (37.2)	313 (25.9)		303 (25.0)	313 (25.9)	
Unmarried and others	2821 (62.8)	897 (74.1)		907 (75.0)	897 (74.1)	
*Race*			< 0.001			0.154
White	3248 (72.3)	813 (67.2)		815 (67.4)	813 (67.2)	
Black	774 (17.2)	201 (16.6)		227 (18.8)	201 (16.6)	
Others	473 (10.5)	196 (16.2)		168 (13.8)	196 (16.2)	
*Histology*			0.529			0.600
SCC	3873 (86.2)	1034 (85.5)		1043 (86.2)	1034 (85.5)	
Non-SCC	622 (13.8)	176 (14.5)		167 (13.8)	176 (14.5)	
*Differentiation*			0.116			0.315
Well or fairly	1607 (35.8)	400 (33.1)		435 (36.0)	400 (33.1)	
Poorly or undifferentiated	1540 (34.2)	414 (34.2)		391 (32.3)	414 (34.2)	
Unknown	1348 (30.0)	396 (32.7)		384 (31.7)	396 (32.7)	
*Tumor size (mm)*			< 0.001			0.186
< 60	1272 (28.3)	393 (32.5)		385 (31.8)	393 (32.5)	
≥ 60	1836 (40.8)	356 (29.4)		396 (32.7)	356 (29.4)	
Unknown	1387 (30.9)	461 (38.1)		429 (35.5)	461 (38.1)	
*2014 FIGO stage*			< 0.001			0.585
IIB	1424 (31.7)	385 (31.8)		380 (31.4)	385 (31.8)	
IIIA	137 (3.0)	89 (7.4)		74 (6.1)	89 (7.4)	
IIIB	2625 (58.4)	620 (51.2)		644 (53.2)	620 (51.2)	
IVA	309 (6.9)	116 (9.6)		112 (9.3)	116 (9.6)	
*Radiotherapy (RT)*			< 0.001			0.870
EBRT alone	2032 (45.2)	676 (55.9)		672 (55.5)	676 (55.9)	
EBRT + BRT	2463 (54.8)	534 (44.1)		538 (44.5)	534 (44.1)	
*Chemotherapy (CT)*			< 0.001			0.963
No/Unknown	408 (9.1)	316 (26.1)		315 (26.0)	316 (26.1)	
Yes	4087 (90.9)	894 (73.9)		895 (74.0)	894 (73.9)	

*SCC* Squamous cell carcinoma, *FIGO* International Federation of Gynecology and Obstetrics, *EBRT* External beam radiotherapy, *BRT* Brachytherapy

### Survival differences between the age groups

The overall 1-, 3-, and 5-year OS rates were 81.0% (95% CI: 0.800–0.820), 55.1% (95% CI: 0.537–0.565) and 47.8% (95% CI: 0.462–0.494), respectively, with a median OS time of 51 months (95% CI: 45.1–56.9) for the entire cohort.

**Fig. 2:**
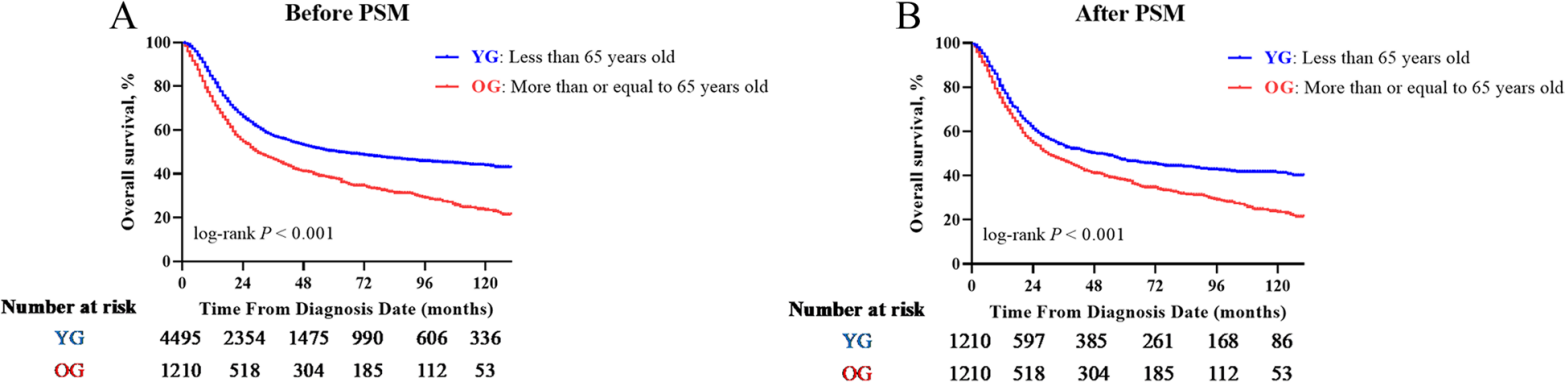
OS comparison according to the age at diagnosis before and after (**A** and **B**) propensity score matching analysis

Before matching, the median OS time was 62.0 months (95% CI: 51.2–72.8), with the 3- and 5-year OS rates of 57.3% (95% CI: 0.557–0.589) and 50.4% (95% CI: 0.486–0.522), respectively, in the YG. The corresponding figures of the median, 3-year, and 5-year OS rates in the OG patients were 30.0 months (95% CI: 25.3–34.7), 46.8% (95% CI: 0.437–0.499), and 38.0% (95% CI: 0.349–0.411) respectively. YG patients had a significantly longer OS outcome than the OG patients (*P* < 0.001, Fig. 2A). To further assess the effect of age on OS, a multivariate analysis by Cox proportional hazards model was performed (Table 2). In the multivariate analysis, the significant variables were as follows: age at diagnosis (< 65 years versus ≥ 65 years, *P* < 0.001, HR = 1.631, 95% CI: 1.307–1.566), marital status (married versus unmarried and others, *P* < 0.001, HR = 1.178, 95% CI: 1.083–1.282), tumor histology (SCC versus Non-SCC, *P* < 0.001, HR = 1.406, 95% CI: 1.268–1.559), tumor differentiation (well or fairly differentiated versus poorly or undifferentiated, *P* < 0.001, HR = 1.211, 95% CI: 1.105–1.328), tumor size (< 60 mm versus ≥ 60 mm, *P* < 0.001, HR = 1.241, 95% CI: 1.122–1.372; < 60 mm versus unknown, *P* < 0.001, HR = 1.302, 95% CI: 1.177–1.441), 2014 FIGO stage (IIB versus IIIA, *P* < 0.001, HR = 1.529, 95% CI: 1.253–1.866; IIB versus IIIB, *P* < 0.001, HR = 1.881, 95% CI: 1.712–2.067; IIB versus IVA, *P* < 0.001, HR = 3.054, 95% CI: 2.645–3.526), RT (EBRT alone versus EBRT + BRT, *P* < 0.001, HR = 0.648, 95% CI: 0.598–0.701) and CT (None/unknown versus Yes, *P* < 0.001, HR = 0.605, 95% CI: 0.545–0.671).

**Table 2:** Univariate and multivariate analyses of OS in stage IIB-IVA cervical carcinoma patients before and after PSM

	Overall survival (Before matching)								Overall survival (After matching)							
	Univariate				Multivariate				Univariate				Multivariate			
	*P value*	HR	*95% CI*	*95% CI*	*P value*	HR	*95% CI*	*95% CI*	*P value*	HR	*95% CI*	*95% CI*	*P value*	HR	*95% CI*	*95% CI*
*Factor*			*Lower*	*Upper*			*Lower*	*Upper*			*Lower*	*Upper*			*Lower*	*Upper*
*Age at diagnosis (years)*																
< 65	1 (reference)				1 (reference)				1 (reference)				1 (reference)			
≥ 65	< 0.001	1.550	1.421	1.691	< 0.001	1.431	1.307	1.566	< 0.001	1.368	1.224	1.529	< 0.001	1.496	1.336	1.675
*Marital status*																
Married	1 (reference)				1 (reference)				1 (reference)				1 (reference)			
Unmarried and others	< 0.001	1.294	1.192	1.405	< 0.001	1.178	1.083	1.282	0.001	1.251	1.098	1.425	0.049	1.143	1.001	1.305
*Race*																
White	1 (reference)				1 (reference)				1 (reference)				1 (reference)			
Black	0.017	1.128	1.022	1.246	0.548	1.031	0.932	1.141	0.030	1.170	1.015	1.348	0.654	1.034	0.894	1.196
Others	0.008	0.841	0.739	0.956	0.054	0.880	0.773	1.002	0.016	0.811	0.684	0.961	0.219	0.898	0.756	1.066
*Histology*																
SCC	1 (reference)				1 (reference)				1 (reference)				1 (reference)			
Non-SCC	< 0.001	1.375	1.241	1.522	< 0.001	1.406	1.268	1.559	< 0.001	1.327	1.144	1.539	< 0.001	1.383	1.190	1.606
*Differentiation*																
Well or fairly differentiated	1 (reference)				1 (reference)				1 (reference)				1 (reference)			
Poorly or undifferentiated	< 0.001	1.238	1.130	1.356	< 0.001	1.211	1.105	1.328	0.003	1.223	1.070	1.397	0.002	1.234	1.079	1.411
Unknown	0.191	1.067	0.968	1.175	0.993	1.000	0.906	1.102	0.803	0.982	0.855	1.129	0.334	0.933	0.811	1.074
*Tumor size (mm)*																
< 60	1 (reference)				1 (reference)				1 (reference)				1 (reference)			
≥ 60	< 0.001	1.350	1.222	1.490	< 0.001	1.241	1.122	1.372	< 0.001	1.443	1.248	1.668	0.013	1.207	1.041	1.400
Unknown	< 0.001	1.493	1.351	1.650	< 0.001	1.302	1.177	1.441	< 0.001	1.462	1.273	1.679	0.018	1.187	1.030	1.367
*FIGO stage (2014)*																
IIB	1 (reference)				1 (reference)				1 (reference)				1 (reference)			
IIIA	< 0.001	1.827	1.499	2.227	< 0.001	1.529	1.253	1.866	< 0.001	1.695	1.338	2.148	< 0.001	1.541	1.215	1.955
IIIB	< 0.001	1.889	1.720	2.074	< 0.001	1.881	1.712	2.067	< 0.001	1.799	1.571	2.059	< 0.001	1.763	1.538	2.022
IVA	< 0.001	3.675	3.191	4.232	< 0.001	3.054	2.645	3.526	< 0.001	3.112	2.568	3.771	< 0.001	2.801	2.298	3.414
*RT*																
EBRT alone	1 (reference)				1 (reference)				1 (reference)				1 (reference)			
EBRT + BRT	< 0.001	0.557	0.516	0.601	< 0.001	0.648	0.598	0.701	< 0.001	0.542	0.483	0.609	< 0.001	0.635	0.563	0.715
*CT*																
No/Unknown	1 (reference)				1 (reference)				1 (reference)				1 (reference)			
Yes	< 0.001	0.505	0.456	0.558	< 0.001	0.605	0.545	0.671	< 0.001	0.546	0.485	0.614	< 0.001	0.577	0.510	0.652

*HR* Hazard ratio, *CI* Confidence interval

Within the propensity-score matched cohort, the median OS time was 49.0 months (95% CI: 36.2–61.8) with the 3- and 5-year OS rates being 53.6% (95% CI: 0.505–0.567) and 46.9% (95% CI: 0.438–0.500), respectively, in the YG. Further, a significant difference in the OS (*P* < 0.001) was reconfirmed after the PSM analysis, as illustrated in Fig. 2B. In the multivariate Cox regression analysis among matched cohorts (Table 2), the significant variables associated with OS were consistent with the Cox regression model obtained before PSM analysis: age at diagnosis, marital status, tumor histology, tumor differentiation, tumor size, 2014 FIGO stage, RT, and CT.

### Subgroup analysis

**Fig. 3:**
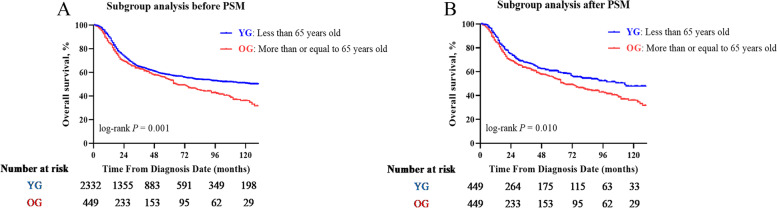
OS comparison according to the age before and after (**A** and **B**) propensity score matching analysis in patients who received trimodal therapy

Since CCRT plus BRT is recommended as the standard treatment option for CC patients with stage IIB-IVA diseases, we conducted a subgroup analysis for patients who underwent a trimodal therapy of EBRT, BRT and CT. There was a significant difference between the proportion of YG and OG patients receiving trimodal therapy (51.9% (2332/4495) and 37.1% (449/1210), respectively; *P* < 0.001). The baseline characteristics of patients of the different age groups who received trimodal therapy are described in Table S1. A good balance in baseline characteristics was achieved after PSM analysis (*n* = 449 in each group). The Kaplan–Meier survival curves for OS in different age groups are illustrated in Fig. 3 A, B. Log-rank tests also revealed a statistically significant difference in OS rates between the group comparison before and after matching (*P* = 0.001 and 0.010, respectively). In the multivariate analysis before PSM, the advanced age at diagnosis was a significant covariate associated with a decreased OS outcome (< 65 years versus ≥ 65 years, *P* < 0.001, HR = 1.362, 95% CI: 1.164–1.594). Even after matching, the advanced age at diagnosis remained as a significant prognostic factor associated with a decreased OS (< 65 years versus ≥ 65 years, *P* = 0.003, HR = 1.377, 95% CI: 1.116–1.699; Table S2).

## Discussion

In this study, we first observed a significant difference in treatment between the two age groups, with the elderly CC patients receiving less BRT, CT, or a combination therapy of CCRT plus BRT than the younger patients. In addition, survival analysis demonstrated that elderly patients had significantly decreased OS time compared to younger patients. Lastly, the Cox regression model and the subgroup comparison for patients who underwent trimodal therapy showed that the advanced age at diagnosis was an independent prognostic factor for decreased OS, both before and after PSM.

As aforementioned, a systemic review has reported the patterns of care for elderly CC patients from 1949 to 2016 [9]. Upon analyzing 24 studies, technical reasons, comorbidities, and the patient’s refusal, including fear of sequelae, came out as the three main reasons for elderly patients not receiving BRT. Although considerable bias existed in this review, still treatment dilemmas among the elderly CC patients need more attention and improvement. Another SEER study with 28,902 CC patients from 1988 to 2005 demonstrated that elderly CC patients were less likely to receive surgery, have adjuvant RT, or BRT. Even after adjusting for treatment disparities, cancer-related mortality was significantly higher in older patients than in younger patients (*P* < 0.001) [29]. Similar findings were also reported in a latter SEER study with patients analyzed between 1988 and 2010 [30].

However, different opinions regarding RT, especially BRT, for elderly CC patients have also been reported [14]. In this study, the authors retrospectively reviewed 113 elderly CC patients who received conventional RT and low dose-rate BRT (LDR-BRT). Grade III-IV rectal complications were observed in two (1.8%) patients, while three (2.7%) patients developed severe urinary tract complications. Only one patient died of acute toxicity due to major diarrhoea. Additionally, EBRT and BRT treatment achieved satisfactory survival outcomes. Hence, the authors strongly supported that BRT should be routinely considered whenever possible for CC patients. Similar findings for elderly CC patients who underwent high dose-rate BRT (HDR-BRT) was reported earlier, where no significant difference was observed upon comparing grade III-IV rectal complications (*P* = 0.12), urinary tract complications (*P* = 0.39), and small bowel complications (*P* = 0.34) with younger patients [31].

Survival benefit, especially OS, is another concern for elderly CC patients who receive standard treatment. In 2017, Wang et al. reported their single center experience of definitive RT or CCRT in 73 elderly CC patients [15]. Among them, 52 (71.2%) patients were diagnosed with FIGO IIB-IVA diseases. The 3-year OS rate was 64.9% for the entire cohort with no treatment-related death. Recently, You et al. also reported their findings from two institutions for elderly CC patients who underwent CCRT in China [32]. They reported that 82 out of 138 patients (59.4%) who were 65 years old were diagnosed with IIB-IIIA diseases. The 5-year OS rate in the CCRT group exceeded 80.0%. Compared to the survival results in the trimodal therapy analysis in the current study, the OG patients had 3- and 5-year OS rates of 63.2% and 53.8%, respectively. Over half of the patients (51.2%) were diagnosed with stage IIIB diseases was a main factor to explain this disparity. Additionally, as for the quality of life (QoL), only a few studies have reported relevant results for elderly CC patients who underwent RT. A study evaluated the correlation between age and QoL among 173 CC patients receiving different treatments, including surgery, RT, and/or CT [33]. The study demonstrated that advanced age at diagnosis (≥ 65 years old) had a significant negative impact on the scores of QoL, which further enhances the importance of paying special attention to elderly CC patients.

Since a vast heterogeneity may exist in elderly CC patients diagnosed with stage IIB-IVA diseases, chronological age alone is a poor descriptor to exclude CC patients from standard treatment. However, progress in this area is still lacking. In a retrospective study from the south-eastern Netherlands [34], they collected the comorbidity data of CC patients. Through multivariate analysis, they demonstrated that heavy comorbidities and advanced tumor stages are independent prognostic factors for impaired OS. Another study evaluated the influence of comorbidity on endometrial cancer (EC) patients treated by adjuvant EBRT and HDR-BRT. This treatment combination was well tolerated in elderly EC patients with good performance and low comorbidity profile [35]. Currently, the NCCN and the International Society of Geriatric Oncology both recommends a comprehensive geriatric assessment (GA), which includes items like functional status, cognition and psychological status, nutrition, polypharmacy, and geriatric syndromes applied in daily clinical decision-making for older cancer patients [36, 37]. In this regard, an international survey focusing on treatment adjustments according to age and frailty status for older CC patients was recently conducted across Europe [38]. Results were partly consistent with previous studies since the treatment modalities and intensity were different for the old and unfit CC patients compared to the young and clinical fit patients. However, the criteria for distinguishing the “unfit” from the “fit” older patients remain uncertain. NCT 02,003,430 was one of the few trials designed to use GA tools, like the Instrumental Activities of Daily Living questionnaire for the pre-treatment evaluation in elderly non-ovarian gynaecological cancers in the USA, and the final results are still awaited.

Unfortunately, our present study is firstly limited by its inherent retrospective analysis during an extensive time frame (from 2004 to 2016). Secondly, critical information like the patients’ baseline characteristics, GA-related domains including haemoglobin levels, socioeconomic status, comorbidities, general performances, nutritional status, geriatric syndromes, psychological status, and staging methods by radiological or surgical approaches, are not available in the SEER database. Thirdly, treatment-related parameters might vary during this study period, like EBRT patterns (Three-dimensional Conformal RT, Intensity Modulated RT, or Volumetric Modulated Arc Therapy), EBRT and BRT dosages and duration, and concurrent CT regimens are also unknown, which might lead to a bias in the final inferences. Finally, whether the findings generated in the SEER database apply to other populations, such as that of developing countries, need to be confirmed in future.

## Conclusions

The present study aimed to evaluate the impact of age at diagnosis on treatment modalities and OS outcomes of stage IIB-IVA CC patients who received RT in the SEER database between 2004 and 2016. We observed that compared with young patients, elderly CC patients (aged ≥ 65 years old) are less likely to receive BRT, CT and trimodal therapy. Through PSM analysis, we further demonstrated that advanced age at diagnosis is associated with significantly decreased OS in these patients. With a growing number of CC patients would being diagnosed at an older age, a comprehensive GA should be taken into consideration to select the appropriate treatment for stage IIB-IVA CC patients in the future.

## Supplementary Information

**Additional file 1:** **Table S1.** Baseline characteristics of patients who received trimodal therapy before and after PSM. **Table S2.** Univariate and multivariate analyses of OS in stage IIB-IVA patients who received trimodal therapy before and after PSM.

## Data Availability

The datasets generated for this study are available on request to the corresponding author.
