# Supplementary material for: Stage IIB-IVA cervix carcinoma in elderly patients treated with radiation therapy: a longitudinal cohort study by propensity score matching analysis
Source: BMC Womens Health. 2023 May 17;23:270. doi: 10.1186/s12905-023-02427-8 (PMC10193738; doi:10.1186/s12905-023-02427-8)
Supplement: Supplementary file 1 — Additional file 1: Table S1. Baseline characteristics of patients who received trimodal therapy before and after PSM. Table S2. Univariate and multivariate analyses of OS in stage IIB-IVA patients who received trimodal therapy before and after PSM. [file 12905_2023_2427_MOESM1_ESM.docx]

**Supplementary material**

Table S1. Baseline characteristics of patients who received trimodal therapy before and after PSM.

| Characteristic | Before matching | | |  | After matching | | |
| --- | --- | --- | --- | --- | --- | --- | --- |
|  | < 65 (n, %) | ≥ 65 (n, %) | P value |  | < 65 (n, %) | ≥ 65 (n, %) | P value |
| *Marital status* |  |  | 0.001 |  |  |  | 0.886 |
| Married | 941 (40.4) | 143 (31.8) |  |  | 145 (32.3) | 143 (31.8) |  |
| Unmarried and others | 1391 (59.6) | 306 (68.2) |  |  | 304 (67.7) | 306 (68.2) |  |
| *Race* |  |  | < 0.001 |  |  |  | 0.855 |
| White | 1731 (74.2) | 311 (69.3) |  |  | 310 (69.0) | 311 (69.3) |  |
| Black | 337 (14.6) | 57 (12.7) |  |  | 62 (13.8) | 57 (12.7) |  |
| Others | 264 (11.2) | 81 (18.0) |  |  | 77 (17.2) | 81 (18.0) |  |
| *Histology* |  |  | 0.107 |  |  |  | 0.371 |
| SCC | 2034 (87.2) | 379 (84.4) |  |  | 369 (82.2) | 379 (84.4) |  |
| Non-SCC | 298 (12.8) | 70 (15.6) |  |  | 80 (17.8) | 70 (15.6) |  |
| *Differentiation* |  |  | 0.602 |  |  |  | 0.737 |
| Well or fairly | 839 (36.0) | 151 (33.6) |  |  | 158 (35.2) | 151 (33.6) |  |
| Poorly or undifferentiated | 812 (34.8) | 159 (35.4) |  |  | 148 (33.0) | 159 (35.4) |  |
| Unknown | 681 (29.2) | 139 (31.0) |  |  | 143 (31.8) | 139 (31.0) |  |
| *Tumor size (mm)* |  |  | < 0.001 |  |  |  | 0.815 |
| < 60 | 699 (30.0) | 172 (38.3) |  |  | 164 (36.5) | 172 (38.3) |  |
| ≥ 60 | 962 (41.2) | 142 (31.6) |  |  | 150 (33.4) | 142 (31.6) |  |
| Unknown | 671 (28.8) | 135 (30.1) |  |  | 135 (30.1) | 135 (30.1) |  |
| *2014 FIGO stage* |  |  | 0.001 |  |  |  | 0.064 |
| IIB | 830 (35.6) | 167 (37.2) |  |  | 176 (39.2) | 167 (37.2) |  |
| IIIA | 58 (2.5) | 24 (5.3) |  |  | 9 (2.0) | 24 (5.3) |  |
| IIIB | 1362 (58.4) | 234 (52.2) |  |  | 237 (52.8) | 234 (52.2) |  |
| IVA | 82 (3.5) | 24 (5.3) |  |  | 27 (6.0) | 24 (5.3) |  |

Table S2. Univariate and multivariate analyses of OS in stage IIB-IVA patients who received trimodal therapy before and after PSM.

|  | Overall survival (Before PSM) | | | | | | | | |  | Overall survival (After PSM) | | | | | | | | |
| --- | --- | --- | --- | --- | --- | --- | --- | --- | --- | --- | --- | --- | --- | --- | --- | --- | --- | --- | --- |
|  | Univariate | | | |  | Multivariate | | | |  | Univariate | | | |  | Multivariate | | | |
|  | ***P*** *value* | HR | *95% CI* | *95% CI* |  | ***P*** *value* | HR | *95% CI* | *95% CI* |  | ***P*** *value* | HR | *95% CI* | *95% CI* |  | ***P*** *value* | HR | *95% CI* | *95% CI* |
| *Factor* |  |  | *Lower* | *Upper* |  |  |  | *Lower* | *Upper* |  |  |  | *Lower* | *Upper* |  |  |  | *Lower* | *Upper* |
| *Age at diagnosis (years)* |  |  |  |  |  |  |  |  |  |  |  |  |  |  |  |  |  |  |  |
| < 65 | 1 (reference) |  |  |  |  | 1 (reference) |  |  |  |  | 1 (reference) |  |  |  |  | 1 (reference) |  |  |  |
| ≥ 65 | 0.001 | 1.293 | 1.107 | 1.510 |  | < 0.001 | 1.362 | 1.164 | 1.594 |  | 0.010 | 1.315 | 1.068 | 1.619 |  | 0.003 | 1.377 | 1.116 | 1.699 |
| *Marital status* |  |  |  |  |  |  |  |  |  |  |  |  |  |  |  |  |  |  |  |
| Married | 1 (reference) |  |  |  |  | - |  |  |  |  | 1 (reference) |  |  |  |  | - |  |  |  |
| Unmarried and others | 0.052 | 1.132 | 0.999 | 1.283 |  |  |  |  |  |  | 0.230 | 1.147 | 0.917 | 1.434 |  |  |  |  |  |
| *Race* |  |  |  |  |  |  |  |  |  |  |  |  |  |  |  |  |  |  |  |
| White | 1 (reference) |  |  |  |  | 1 (reference) |  |  |  |  | 1 (reference) |  |  |  |  | - |  |  |  |
| Black | 0.847 | 0.983 | 0.827 | 1.169 |  | 0.826 | 1.020 | 0.857 | 1.213 |  | 0.349 | 0.861 | 0.629 | 1.178 |  |  |  |  |  |
| Others | 0.007 | 0.756 | 0.616 | 0.927 |  | 0.018 | 0.780 | 0.635 | 0.958 |  | 0.068 | 0.758 | 0.563 | 1.021 |  |  |  |  |  |
| *Histology* |  |  |  |  |  |  |  |  |  |  |  |  |  |  |  |  |  |  |  |
| SCC | 1 (reference) |  |  |  |  | 1 (reference) |  |  |  |  | 1 (reference) |  |  |  |  | 1 (reference) |  |  |  |
| Non-SCC | < 0.001 | 1.500 | 1.277 | 1.764 |  | < 0.001 | 1.482 | 1.258 | 1.744 |  | 0.003 | 1.474 | 1.144 | 1.899 |  | 0.002 | 1.504 | 1.164 | 1.945 |
| *Differentiation* |  |  |  |  |  |  |  |  |  |  |  |  |  |  |  |  |  |  |  |
| Well or fairly differentiated | 1 (reference) |  |  |  |  | 1 (reference) |  |  |  |  | 1 (reference) |  |  |  |  | - |  |  |  |
| Poorly or undifferentiated | < 0.001 | 1.296 | 1.124 | 1.495 |  | 0.004 | 1.234 | 1.069 | 1.425 |  | 0.930 | 1.011 | 0.791 | 1.293 |  |  |  |  |  |
| Unknown | 0.403 | 1.069 | 0.915 | 1.249 |  | 0.511 | 1.054 | 0.901 | 1.234 |  | 0.271 | 0.865 | 0.668 | 1.120 |  |  |  |  |  |
| *Tumor size (mm)* |  |  |  |  |  |  |  |  |  |  |  |  |  |  |  |  |  |  |  |
| < 60 | 1 (reference) |  |  |  |  | 1 (reference) |  |  |  |  | 1 (reference) |  |  |  |  | 1 (reference) |  |  |  |
| ≥ 60 | < 0.001 | 1.349 | 1.158 | 1.572 |  | 0.002 | 1.274 | 1.092 | 1.487 |  | < 0.001 | 1.619 | 1.254 | 2.090 |  | 0.011 | 1.413 | 1.083 | 1.843 |
| Unknown | < 0.001 | 1.390 | 1.185 | 1.629 |  | < 0.001 | 1.345 | 1.146 | 1.578 |  | 0.031 | 1.335 | 1.027 | 1.735 |  | 0.143 | 1.220 | 0.935 | 1.593 |
| *FIGO stage (2014)* |  |  |  |  |  |  |  |  |  |  |  |  |  |  |  |  |  |  |  |
| IIB | 1 (reference) |  |  |  |  | 1 (reference) |  |  |  |  | 1 (reference) |  |  |  |  | 1 (reference) |  |  |  |
| IIIA | 0.011 | 1.572 | 1.111 | 2.224 |  | 0.026 | 1.486 | 1.049 | 2.105 |  | 0.010 | 1.972 | 1.178 | 3.301 |  | 0.012 | 1.956 | 1.162 | 3.292 |
| IIIB | < 0.001 | 1.816 | 1.580 | 2.087 |  | < 0.001 | 1.807 | 1.571 | 2.079 |  | < 0.001 | 1.983 | 1.560 | 2.521 |  | < 0.001 | 1.911 | 1.500 | 2.434 |
| IVA | < 0.001 | 2.998 | 2.244 | 4.004 |  | < 0.001 | 2.801 | 2.094 | 3.748 |  | < 0.001 | 3.715 | 2.463 | 5.603 |  | < 0.001 | 3.431 | 2.248 | 5.237 |

HR, hazard ratio; CI, confidence interval.
